# Changes in Tendon Thickness After Chondrovita FIT^®^ Supplementation in Elite Skaters: Findings from a Quasi-Experimental Study

**DOI:** 10.3390/ijerph22010024

**Published:** 2024-12-28

**Authors:** Silvana Giannini, Stefano Amatori, Mario Vetrano, Michela Battistelli, Annalisa Belli, Giorgia Simona Musicco, Marco Bruno Luigi Rocchi, Davide Sisti, Fabrizio Perroni

**Affiliations:** 1 Villa Stuart Sport Clinic-FIFA Medical Centre of Excellence, 00136 Rome, Italy; dr.silvana.giannini@gmail.com; 2 Department of Biomolecular Sciences, Section of Exercise and Health Sciences, University of Urbino Carlo Bo, 61029 Urbino, Italy; stefano.amatori@uniecampus.it (S.A.); michela.battistelli@uniurb.it (M.B.); g.musicco@campus.uniurb.it (G.S.M.); fabrizio.perroni@uniurb.it (F.P.); 3 Department of Theoretical and Applied Sciences, eCampus University, Novedrate, 22060 Como, Italy; 4 Physical Medicine and Rehabilitation Unit, Sant’Andrea Hospital, “Sapienza” University of Rome, 00189 Rome, Italy; mario.vetrano@uniroma1.it; 5 Department of Biomolecular Sciences, Service of Biostatistics, University of Urbino Carlo Bo, Piazza Rinascimento 7, 61029 Urbino, Italy; marco.rocchi@uniurb.it (M.B.L.R.); davide.sisti@uniurb.it (D.S.); 6 “Museum of Football F.I.G.C.” Foundation, Italian Football Federation, Via Gregorio Allegri 14, 00198 Rome, Italy

**Keywords:** athletes, collagen hydrolysate, injury prevention, sports nutrition, tendon thickness, ultrasound assessment

## Abstract

The use of dietary supplements is widespread in sports and fitness, with many products containing multiple ingredients. Among supplements often consumed to support musculotendinous health, collagen hydrolysate (CH) has gained popularity for its potential in improving joint comfort and function. This single-blind quasi-experimental study investigated the effects of a three-month oral supplementation with a specific CH-based product, Chondrovita FIT^®^ (Bone Srl, Rome, Italy), on tendon structure in elite Italian skaters. Eighteen male and female elite skaters (mean age: 21 ± 3 years) participated, receiving daily pre-workout (4500 mg CH) and post-workout (2500 mg CH) doses. Tendon structure in the patellar and peroneal tendons was assessed using ultrasound imaging at baseline and post-supplementation. Results showed a significant increase in tendon thickness in both the patellar and peroneal tendons after supplementation, although no changes were observed in the tendon cross-sectional area. These findings suggest that Chondrovita FIT^®^ supplementation may induce beneficial structural changes in tendons, potentially supporting tendon health and performance in high-load sports. However, further research is needed to confirm long-term effects and functional outcomes.

## 1. Introduction

The use of dietary supplements in sports and fitness is widespread, with reasons ranging from enhancing performance (e.g., ergogenic aids) to promoting health (e.g., vitamins) [1,2]. Often, supplements are combined to achieve greater performance benefits, though little is known about the interactions between different supplements taken simultaneously, despite speculation that some combinations may have synergistic effects [3]. In addition, many supplements are often marketed as “multi-ingredient performance supplements” (MIPS). For example, pre-workout blends often contain caffeine, creatine, beta-alanine, and other ingredients. Similarly, post-workout supplements—designed to support recovery and muscle repair—typically include protein, branched-chain amino acids (BCAAs), creatine, and more [4,5]. Although in some studies the presence of a multi-ingredient composition may be considered a limitation, as it does not allow to determine the relative impact of each component on the outcome, investigating them in real-world settings is essential since they are frequently consumed by both athletes (e.g., due to sponsorship) and the general population.

Among the supplements frequently consumed to improve musculotendinous health and adaptation processes, collagen hydrolysate (CH)—a food ingredient that has the potential to improve articular well-being and function—has gained popularity [6]; The International Olympic Committee (IOC) Consensus Statement on dietary supplements for high-performance athletes [7] also suggests that CH-containing supplements could be beneficial for athletic populations. Collagen is a natural component of the diet, found in animal products such as meat and fish. The CH is absorbed in the intestine [8], is especially contained in fermented dairy products [9], has been used for many years (as gelatin in foods), and has been declared safe [10], making CH an ideal ingredient for functional food. Collagen contains unique amino acids not found in any other protein, namely, hydroxyproline, and hydroxylysine, which could help maintain the structure and function of the articular cartilage, thus improving articular well-being safely and effectively [11]. CH has been shown in vitro to significantly increase the biosynthesis of type II collagen in chondrocytes in bovine [12] and human [13] cell cultures and to significantly increase the biosynthesis of proteoglycans in chondrocytes in humans. Numerous studies [6,14,15,16,17,18] have shown that a daily intake of 10 g of CH for 60 days or more led to a reduction in pain in patients with hip or knee osteoarthritis, believing that this is due to a specific effect of CH on joint tissues. Studies by Trč and Bohmová [19] and Moskowitz [6] showed a statistically significant reduction in pain, a decrease in analgesic consumption, and improved mobility in patients with hip or joint arthritis who received a daily dose of 10 g of CH for at least three months, while Crowley et al. [20] showed a significant improvement in daily activities, suggesting an improvement in quality of life in patients with knee osteoarthritis following a daily supplementation of 40 mg type II collagen for 90 days. Despite the well-established relationship between exercise stimulus/loading and collagen synthesis [21,22], research on the role of supplements that support these adaptations is in its early stages. Clinical trials [23,24] showed improved joint pain in athletes who complained about joint pain or discomfort and were treated with the dietary supplement CH. Recent studies [25,26] showed that vitamin C-enriched CH supplementation had a promising effect on the recovery of jumpers’ knees and improved the rate of force development in the squat and counter-movement jump alongside training.

In this context, figure skating is a sport where CH supplementation may be particularly beneficial for athletes’ joint health. Competitive figure skating is highly complex, requiring both endurance and strength; skaters must perform challenging elements, such as jumps, spins, and other choreographed movements [27]. As a result, these athletes need to achieve high technical proficiency and strength to execute their demanding routines while maintaining an aesthetically pleasing appearance [27]. Due to the intense training involved in perfecting increasingly difficult skills, figure skaters are at risk of chronic overuse injuries, predominantly in the lower extremities. Ankles and knees are the most commonly injured joints, with tendinopathies being particularly frequent [28], as frequently reported in other sports involving repetitive jumping and landing mechanics [26]. Previous research showed that pre-exercise supplementation with 15 g of CH combined with 50 mg of vitamin C led to increased collagen synthesis [29], which appears necessary for enhancing tendon mechanical properties in response to exercise [30].

Given this context, the present quasi-experimental study aimed to assess changes in tendon structural parameters in elite figure skaters using ultrasound measurements after three months of a multi-ingredient CH-based oral supplementation, Chondrovita FIT^®^. We hypothesized that improved tendon and myofascial tropism would result in enhanced joint structure.

## 2. Materials and Methods

### 2.1. Study Design

This study had a single-blind quasi-experimental design. Although the absence of a control group represents a limitation, previous studies have reported no changes in tendon thickness in response to training programs of different types and durations [31,32,33]. Consequently, while acknowledging this limitation, we considered the effect of training on tendon thickness to be negligible, justifying our choice of a quasi-experimental design.

The patellar and peroneal tendons of eighteen elite skaters were evaluated via ultrasound techniques at the end of the competitive season (i.e., September) and three months after (i.e., December) the supplementation period with Chondrovita FIT^®^ (Chondrovita, Bone Srl, Rome, Italy) (see Section 2.3 for the supplement’s detailed composition). This period corresponded to the transition phase of the annual preparation, as it was right after the main competitions (European Championships in August, World Championships in September). The study was in accordance with the ethical standards of the institutional and national research committee and with the 1964 Helsinki Declaration and its later amendments, and it was approved by the Ethics Committee of the University of Urbino (no. 75/26.10.2023). Before the day of the study and after a verbal and written explanation of the experimental design of the nature of the study, a signed consent form was filled out by athletes (>18 years) or their parents (if <18 years). In addition, the researcher informed that each subject could withdraw from the study at any time.

Once they arrived at the clinic center, each participant was conducted to the medical room, where their anthropometric measurements were collected. Body mass (kg) and height (m) were measured in light clothes and barefoot using an electronic scale (±0.1 kg) and fixed stadiometer (±0.1 cm) (Seca 702, Seca GmbH & Co. KG, Hamburg, Germany). Following this, the participants were then given the supply of supplements for the next three months, and detailed guidance on how and when to take them was provided, in accordance with the manufacturer’s guidelines. During the three months of supplementation, participants followed their habitual diet, provided by a sport nutritionist, and training regimens. Although the training was not directly monitored, the athletes verbally reported training six days per week, with an average duration of three hours for each training session.

### 2.2. Participants

Eighteen Italian male (n = 9) and female (n = 9) elite skaters (age: 21 ± 3 years; height: 1.68 ± 0.13 m; body mass: 65.9 ± 11.2 kg; BMI: 23.1 ± 1.1 kg·m^−2^) voluntarily participated to the study. The participants were classified as Tier 4 (elite/international level) and Tier 5 (world-class), according to the classification proposed by McKay et al. [34]. Individuals were recruited as elite healthy athletes without compliance about joint pain or joint discomfort due to joint stress, injury, surgical outcome, or trauma. Subjects were included if there was no indication of structural issues by a former clinical diagnosis or the physician’s assessment during the initial visit. The exclusion criteria were muscle-tendon pathologies (surgical treatments of the tendons and joints in question, previous intra-articular fractures occurring in the last three years), endocrine, gastrointestinal, respiratory, hepatic, renal, or metabolic diseases, steroid intake in the 7 days before enlistment; hypersensitivity reactions to protein products; unavailability or inability to comply with study procedures. One participant retired from the study due to personal reasons; the analyses then included 17 participants (9 males, 8 females).

### 2.3. Product Description

Chondrovita FIT^®^ product (Chondrovita, Bone Srl, Rome, Italy) is composed of two different preparations (green and blue) to be taken at different training times (green: pre-workout; blue: post-workout), which contain 4500 mg and 2500 mg of CH, respectively. The boxes containing the product carried neither the commercial name of the product nor its detailed contents, so as to limit the placebo effect on the effectiveness of the supplement itself. The participants were then blinded with respect to the treatment. The type I collagen contained in Chondrovita FIT^®^ is produced by Gelita^®^ (Gelita Deutschland GmbH, Eberbach, Deutschland) and is derived from animal collagen, which undergoes enzymatic hydrolysis followed by filtration and purification. It consists of short-chain peptides: chromatographic analysis has shown that their molecular weight ranges from 0.5 to 15 kDa, with an average molecular weight of approximately 3 kDa. The high bioavailability of these peptides from CH is precisely due to their small size.

The pre-workout formulation is thought to be taken shortly before the workout by dissolving the sachet in 250 mL of water, as its components could support the body during exercise. The post-workout preparation should be taken on the same day, after the workout, in the evening before bedtime. Its active ingredients help the body recover energy after physical activity, shortening the time needed for natural metabolic and energy restoration, while reducing fatigue and promoting sleep. For example, proteins contribute to muscle growth, maintenance of muscle mass, and preservation of the skeletal system, *Withania somnifera* is an adaptogenic tonic important for combating physical and mental fatigue, and it also promotes physiological relaxation, while Vitamin C is an antioxidant that supports normal bone and cartilage health and energy metabolism. The exact composition of the two preparations is reported in Table 1. The supplement was taken before and after each training session (six training sessions per week for 12 weeks).

### 2.4. Ultrasound Assessment

To reduce measurement variations, ultrasound assessments of patellar and peroneal tendons were all performed by the same medical radiologist, who had more than 30 years of clinical imaging experience for musculoskeletal disorders. Tendons and their area and tendon thicknesses (proximal, medial, and distal) were chosen according to previous studies [35,36,37,38,39,40,41] and measured with a Philips iU22 ultrasound machine with a 17.5 MHz linear array transducer (Philips, Amsterdam, The Netherlands). Skaters were examined in a supine position with the knees flexed (~20°), and gray-scale examinations were performed using 2D with a depth of 18–20 mm, AO = 100%, DR = 70, and gain = 68. To obtain a single image of the entire patellar tendon, longitudinal and axial views of the tendon were obtained by means of a specific panoramic viewing system in the sagittal plane, while anterior–posterior patellar tendon thickness was measured from the superficial to the deep peritenonium, 0.5 cm distal from the apex of the patella, 0.5 cm proximal from the tibial tuberosity, and in the center of these two points. Finally, we performed an axial view to measure the tendon area. The athlete was invited to keep the quadriceps muscle contracted to obtain the echogram in isometric contraction. The peroneal tendons were evaluated with the patient in lateral decubitus with a flexed knee, leg fully supported on the medical table to avoid motion artifacts, and foot in an indifferent position to obtain a good angle for tendon study. Longitudinal-oblique scans were performed along the major axis of the peroneal tendons, and subsequent axial retromalleolar and distal scans were performed to obtain the tendon area.

### 2.5. Treatment Satisfaction

After the supplementation period, the Treatment Satisfaction Questionnaire for Medication (TSQM) [42] was completed. In order to allow participants to respond to the questionnaire with full awareness (as they were blinded until the end of supplementation), a researcher explained the supplement’s contents and its intended function. The researcher was also available to provide any necessary clarification regarding the questionnaire items. The TSQM is a 14-item questionnaire comprising four subscales: effectiveness, convenience, side effects, and global satisfaction, evaluated using a Likert scale. Each subscale had a maximal score of 100, and the subscales were then summed up to obtain an overall satisfaction score (maximum score of 400). The *“Effectiveness”* section comprised three questions regarding the “Ability to prevent or treat condition” (Q1), “Way supplementation relieves symptoms” (Q2), and “Time it takes supplementation to start working” (Q3). The “*Side effects*” section investigated “who reported any side effect” (Q4; dichotomic response: yes or no); if participants answered yes, then the other items of the subscale explored the “Bothersomeness of side effects” (Q5), whether side effects interfered with physical function (Q6), with mental function (Q7) and if they impacted the overall satisfaction (Q8). The *“Convenience”* section focused on “Ease/difficulty to use” (Q9), “Ease/difficulty of planning” (Q10), and “Convenience to take as instructed” (Q11). *The “Global satisfaction”* section explored “Confidence that taking supplements is good” (Q12), “Certainty that good things about supplementation outweigh the bad things” (Q13), and “Satisfaction with supplementation” (Q14).

### 2.6. Statistical Analyses

The statistical analysis was designed to assess changes pre- and post-supplementation and included multiple clinical parameters. Primary outcomes included changes in tendon area and thickness, as measured by ultrasound exam. Secondary outcomes included scores from the TSQM, which assessed patient satisfaction in terms of effectiveness, convenience, global satisfaction, and side effects. Mean values and standard deviations (SD) were calculated for all continuous variables. Violin plots were used to visualize the distribution of variables before and after the intervention, and these graphical tools helped in identifying central tendencies (median) and the shape of dispersion within the data. A general linear model approach was employed to handle the repeated measures data, which included assessments taken at baseline (pre-supplementation) and three months post-supplementation. The model’s main predictors were the tendon type (patellar vs. peroneal) and the time point (pre- vs. post-supplementation). Interaction terms between tendon type and time point were included to investigate if the effect of supplementation varied across different tendons. The partial eta squared (*η*^2^*_p_*) was considered as effect size measure, and interpreted as *η*^2^*_p_* = 0.01: small effect; *η*^2^*_p_* = 0.06: medium effect; *η*^2^*_p_* = 0.14 large effect. All statistical analyses were performed using SPSS (version 22). The significance level was set at *p* < 0.05 for all tests.

## 3. Results

### 3.1. Tendons’ Measurements

In the pre-treatment condition, females tend to have slightly smaller tendons (both in terms of area and thickness) than their male counterparts, although the multivariate model was not statistically significant (*F*_(1,27)_ = 3.178, *p* = 0.086, *η*^2^*_p_* = 0.105). Consequently, we decided not to consider gender as a factor for the subsequent analyses. Table 2 reports the patellar and peroneal tendons’ area and thickness (anteroposterior distance, APD) values.

**Figure 1:**
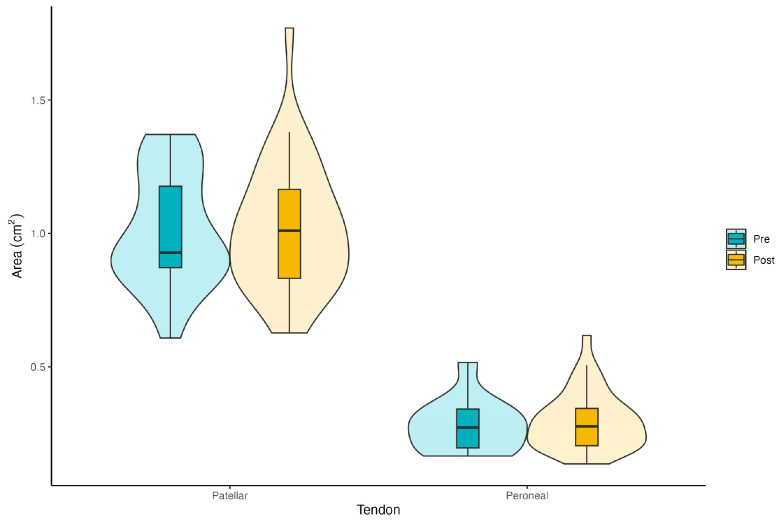
Patellar and peroneal tendons’ area (cm^2^) in the pre- and post-measurements.

The difference in tendon area between pre- and post-treatment was not statistically significant (*F*_(1,38)_ = 0.216, *p* = 0.645, *η*^2^*_p_* = 0.006), suggesting no significant change in tendon area due to the treatment. The tendon factor, representing the difference between tendon types, was highly significant (*F*_(1,38)_ = 107.71, *p* < 0.001, *η*^2^*_p_* = 0.818), indicating a significant difference in area measurements between the patellar and peroneal tendons. The interaction was not significant (*F*_(1,38)_ = 0.122, *p* = 0.980, *η*^2^*_p_* = 0.001), suggesting that the effect of treatment did not differ significantly between the two tendon types. The results are graphically presented in Figure 1.

**Figure 2:**
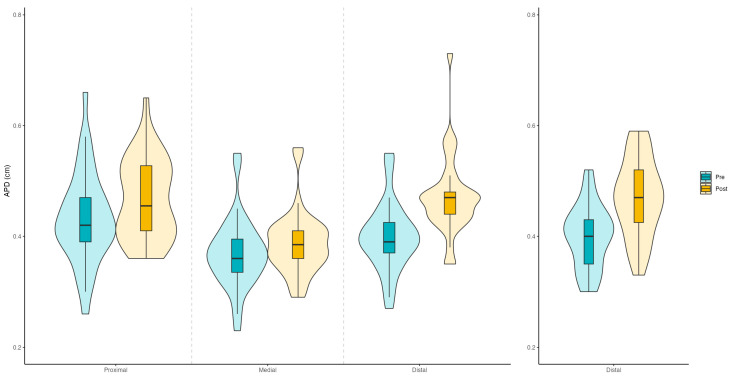
Patellar and peroneal tendons’ thickness (antero-posterior distance, cm), in the pre- and post-measurements. The patellar tendon (**left plots**) was measured in the proximal, medial, and distal sections, while the peroneal tendon (**right plot**) was only in its distal section.

The tendon thickness (anteroposterior distance) was measured in the proximal, medial, and distal sections of the patellar tendons, while only in the distal part of the peroneal tendon. In the proximal and medial sections of the patellar tendon, the treatment effect, representing the difference between pre- and post-treatment, was statistically significant in both sections (proximal: *F*_(1,32)_ = 4.698, *p* = 0.038, *η*^2^*_p_* = 0.128; medial: *F*_(1,32)_ = 7.345, *p* = 0.011, *η*^2^*_p_* = 0.187). Considering the distal section, pre- vs. post-treatment (*F*_(1,56)_ = 85.070, *p* < 0.001, *η*^2^*_p_* = 0.603) was highly significant, suggesting a significant difference in APD measurements before and after treatment; the partial eta squared indicates a large effect size, suggesting a substantial impact of the pre- vs. post-treatment condition on the distal APD measurements. The tendon type was not significant (F_(1,56)_ = 0.001, *p* = 0.977, *η*^2^*_p_* = 0.001), indicating no significant difference in distal APD measurements between the patellar and peroneal tendons, independently of the measurement time. The interaction between time (pre vs. post) and tendon type was not significant (*F*_(1,56)_ = 0.374, *p* = 0.543, *η*^2^*_p_* = 0.007), suggesting that the effect of treatment did not differ significantly between the two tendon types. The results on tendons’ thickness are graphically presented in Figure 2.

### 3.2. Treatment Satisfaction

Male athletes reported higher mean scores in all categories than female counterparts, particularly in effectiveness, convenience, global satisfaction, and side effects, even if the observed differences are minimal and lack practical significance, considering the large standard deviations. Descriptive statistics of TSQM are presented in Table 3.

## 4. Discussion

This quasi-experimental study investigated the effects of Chondrovita FIT^®^ supplementation on tendon structure in elite skaters, providing novel insights into the potential benefits of nutritional interventions for tendon health in high-performance athletes. The primary finding was a significant increase in tendon thickness for both patellar and peroneal tendons after three months of Chondrovita FIT^®^ supplementation, without differences in the tendon area. Additionally, a more pronounced increase was observed in the distal section of both patellar and peroneal tendons, presenting an intriguing pattern of adaptation that warrants further exploration.

The observed increase in tendon thickness may indicate a positive structural adaptation, potentially enhancing the tendon’s capacity to withstand the substantial mechanical loads experienced during skating. This finding aligns with previous research by Schunck et al. [43], who reported improved collagen synthesis in the extracellular matrix of tendons following CH supplementation. Moreover, it extends our understanding of tendon adaptations to nutritional interventions, suggesting that such changes can occur within a relatively short timeframe (three months in our case) in young elite athletes. However, it is crucial to interpret these findings with caution. While a moderate increase in tendon thickness is likely beneficial, excessive thickening could indicate pathological changes such as inflammation or degeneration [44]. The fact that the observed thickening was greater in the distal region of the tendons is particularly interesting. This regional specificity in tendon adaptation has been previously observed in response to exercise interventions [45], but our study is among the first to report such effects with nutritional supplementation. This heterogeneity in tendon response could be particularly relevant for sports-specific adaptations, as different regions of tendons may be subjected to varying stresses depending on the biomechanics of the sport in question, and this finding could have significant implications for understanding the mechanisms of tendon adaptation and developing targeted interventions to enhance tendon health in specific anatomical regions.

The lack of significant changes in the tendon area is consistent with findings from other studies in the literature. For example, Lee et al. [30] reported no changes in patellar tendon cross-sectional area (CSA) after 10 weeks of 30 g CH supplementation (with 500 mg of vitamin C) in female soccer players. Similarly, Balshaw and colleagues [46] observed no improvements in the patellar tendon CSA following 15 weeks of resistance training combined with 15 g of collagen peptides supplementation in young active men. In contrast, Jerger et al. [47] reported increased Achilles tendon CSA after a 14-week high-load resistance training program with daily supplementation of 5 g collagen peptides. These contrasting findings may suggest that the effects of CH supplementation are more pronounced in specific tendons, possibly due to differences in vascularization, load distribution, or metabolic activity. Furthermore, the dose–response relationship should also be considered, as a recent study reported that ingesting 30 g of CH induced a greater acute collagen synthesis response than ingesting 15 g or 0 g of CH [48].

The clinical implications of our findings are manifold and potentially far-reaching. For sports medicine practitioners, the possibility of enhancing tendon structure through nutritional supplementation offers a new avenue for injury prevention and performance optimization. Collagen supplementation could also be potentially beneficial in the treatment of Achilles tendinopathies; although there is currently insufficient evidence to determine the real efficacy of conservative treatments for Achilles tendinopathies [49], collagen supplementation appeared to improve clinical and/or structural outcomes [50]. The potential strengthening of the tendons could be particularly beneficial for skaters who experience high stress on their tendons during rapid accelerations, decelerations, and direction changes. This structural improvement might contribute to reduced injury risk, enhanced force transmission, and potentially improved performance metrics such as speed and agility. It is important to note that ultrasound iconography always showed increased thickness of the tendon and never of the peritenon. The absence of thickening of the peritenonium excludes phenomena that are phlogistic and reactive to overuse. The homogeneity on the ultrasound exam depicts excellent fibrillar enhancement. Moreover, the implications extend beyond skating to other sports involving high tendon loads, such as basketball, volleyball, and track and field events. If corroborated by further research, these findings could inform the development of sport-specific nutritional strategies aimed at enhancing tendon health and resilience. The potential for CH supplementation to accelerate tendon adaptation or support recovery from tendon injuries could have significant implications for rehabilitation protocols and return-to-play decisions. From a broader health perspective, these findings contribute to our understanding of tendon physiology and the potential for nutritional interventions to modulate tendon structure. This could have implications for athletes and the general population, particularly in the context of age-related tendon degeneration or occupational tendon disorders [51].

The study’s strengths include its focus on a specific, elite athletic population and the use of ultrasound for comprehensive tendon assessment. These methodological choices enhance the reliability and relevance of the findings for sports medicine practice. However, several limitations of the study should be acknowledged. The lack of a control group limits our ability to definitively attribute the observed changes to Chondrovita FIT^®^ supplementation alone, as factors such as training effects or seasonal variations cannot be ruled out (despite, as reported in the Methods section, several studies did not report any change in tendon thickness after a training period, in particular in athletes). The three-month intervention period, while sufficient to observe structural changes, may not capture the full extent of long-term adaptations or potential side effects. Furthermore, the lack of a detailed monitoring over food intake would also represent a limitation in the interpretation of our results. Additionally, while valuable, the study’s focus on structural changes does not provide direct evidence of functional improvements or injury risk reduction. Finally, we tested the effect of a multi-ingredient supplement, the Chondrovita FIT^®^, and this likely confers a greater external validity to our study, as athletes are more likely to buy and consume multi-ingredient supplements in the real world (e.g., for sponsorship reasons), but at the same time, this did not allow us to investigate the contribution of the collagen hydrolysate alone on the results.

## 5. Conclusions

This study provides compelling preliminary evidence that Chondrovita FIT^®^ supplementation may induce structural changes in the tendon thickness of elite skaters, but not in the tendon area. These changes could potentially contribute to improved tendon health, enhanced performance, and reduced injury risk, although further research is needed to establish causality and long-term effects. The findings support the hypothesis that CH-based supplementation might be a promising strategy for supporting tendon health in athletes. However, it should be considered part of a comprehensive approach to training, nutrition, and injury prevention. Future studies should address the limitations of this preliminary investigation by including a control group, extending the intervention period, and incorporating food intake, training logs and functional performance measures, and injury surveillance to better understand the clinical significance of these structural changes. Additionally, investigating the mechanisms underlying the regional specificity of tendon adaptation could provide valuable insights for developing more targeted and effective interventions in sports medicine. Furthermore, it is anecdotally known that athletes may consume these products for many years, so more research is needed to investigate the effect of chronic multi-ingredient supplementation consumption on structural adaptations and general health. The potential implications of these findings for both elite athletes and the general population underscore the importance of continued research in this area. As our understanding of the interplay between nutrition and tendon health grows, we may be able to develop more effective strategies for maintaining tendon health across the lifespan, from high-performance sports to healthy aging.

## Figures and Tables

**Table 1:** Chondrovita FIT^®^ pre- and post-workout formulations.

	Pre-Workout	Post-Workout
Collagen hydrolysate	4500 mg	2500 mg
Whey protein	-	2500 mg
Cocoa 22/24	-	1200 mg
Creatine monohydrate	1000 mg	-
L-Leucine	800 mg	800 mg
L-Isoleucine	400 mg	400 mg
L-Valine	400 mg	400 mg
L-Arginine AKG	500 mg	-
Ashwagandha 2.5%	-	150 mg
Vitamin C	80 mg	80 mg
Zinc	50 mg	-
Lactase (2500 FCC)		25 mg
Magnesium	3.75 mg	-
Vitamin B	2.6 mg	1.4 mg

Note: 22/24 = Medium alkalized cocoa powder with 22/24% cocoa butter; Ashwagandha 2.5% = *Whitania somnifera* root extract titled 2.5% whitanolide; AKG = alpha-ketoglutarate; FCC = Food Chemical Codex (measure of enzymatic activity).

**Table 2:** Comparison of pre- and post-treatment measurements of patellar and peroneal tendons’ area and anteroposterior distance (APD) values. Data are shown as mean ± SD.

Tendon	Measure	Pre	Post	Δ%
Patellar	Area (cm^2^)	1.017 ± 0.217	1.035 ± 0.259	+1.77
	APD Proximal (cm)	0.437 ± 0.083	0.463 ± 0.073 *	+5.95
	APD Medial (cm)	0.369 ± 0.065	0.387 ± 0.059 *	+4.88
	APD Distal (cm)	0.396 ± 0.063	0.471 ± 0.072 *	+18.9
Peroneal	Area (cm^2^)	0.279 ± 0.099	0.299 ± 0.095	+7.17
	APD Proximal (cm)	-	-	-
	APD Medial (cm)	-	-	
	APD Distal (cm)	0.391 ± 0.067	0.476 ± 0.072 *	+21.7

*Note:* * significantly different from pre-treatment.

**Table 3:** Descriptive statistics of TSQM responses are presented as mean ± SD [min–max].

	Male (n = 9)	Female (n = 8)
Effectiveness (max. 100)	76 ± 10 [57–86]	68 ± 15 [52–90]
Convenience (max. 100)	80 ± 12 [67–100]	76 ± 19 [52–100]
Global satisfaction (max. 100)	82 ± 13 [56–94]	74 ± 16 [53–94]
Side effect (max. 100)	95	75 ± 14 [65–85]
**Total** **s** **core (max. 400)**	337 ± 21 [292–368]	331 ± 54 [247–385]

## Data Availability

The data that support the findings of this study are available from the corresponding author upon reasonable request.
